# Examining the potential impacts of climate change on international security: EU-Africa partnership on climate change

**DOI:** 10.1186/2193-1801-3-194

**Published:** 2014-04-17

**Authors:** Mahamat K Dodo

**Affiliations:** School of International Studies, Pusan National University, EU Center, Busan, South Korea

**Keywords:** Climate change impacts, Africa, Europe, Development assistance

## Abstract

**Abstract:**

Climate Change like many global problems nowadays is recognized as a threat to the international security and cooperation. In theoretical terms, it is being securitized and included in the traditional security studies. Climate change and its accompanying environmental degradation are perceived to be a threat that can have incalculable consequences on the international community. The consequences are said to have more effects in small island developing nations and Africa where many States are fragile and overwhelmed with mounting challenges. In recent years, the security implications of the climate change are being addressed from national, regional and multilateral level. Against this backdrop, this paper intends to contribute to the debate on climate change and international security and present a broader perspective on the discussion. The paper will draw from the EU-Africa partnership on climate change and is structured as follows: the first part introduces the background of the international climate change policy and its securitization, the second part covers the EU-Africa relations and EU-Africa partnership on climate change, and the third part discusses the Congo Basin Forest Partnership as a concrete example of EU-Africa Partnership on Climate Change. Lastly, the paper concludes by drawing some conclusions and offers some policy perspectives and recommendations.

**JEL classification:**

Q54; 055; 052; 01;

## Introduction

Global climate change challenges have been at the forefront of the multilateral agenda in the last quarter of a century. Beginning with the *Montreal Protocol on Substances that Deplete the Ozone Layer* (Montreal Protocol) of 1987, and the *Rio Conventions* (UN Convention on Biological Diversity, UN Framework Convention on Climate Change, and UN Convention to Combat Desertification) of 1992, climate change issues have become policy area concerns for many governments across the globe. This has been true for larger developing countries, least-developed countries, and industrialized developed ones as well. As such, because of the nature of the global climate change challenges, the international community through the United Nations Framework Convention on Climate Change (UNFCCC) had embarked on comprehensive negotiations aimed at finding adequate solutions to the adaptation and mitigation of the global climate change.

Major industrialized countries like the United States, Canada, England, Germany and France, and many emerging ones such as China, India, Russia, South Africa and Brazil have entered into various coalition and alliances with like-minded countries in the international fora; convened to discuss, debate, and draft agreements to combat the threats of the climate change. In that respect, the United Nations Environment Programme and the World Meteorological Organization established in 1988 the Intergovernmental Panel on Climate Change (IPCC). They entrusted it with a mandate to provide the international community with technical and up to date scientific information on climate change and its effects on the world at large. Since then, the various reports provided by the IPCC have served as guiding frameworks through which governments around the world adopt and respond to the issues of climate change that their respective societies face.

Subsequently, in 1997, the world community adopted an international agreement known as the Kyoto Protocol (KP) at Kyoto, Japan. This accord entered into force in 2005 and was linked with the United Nations Framework Convention on Climate Change. The main feature of the agreement was that it set binding targets for the European Community and 37 major industrialized nations to reduce their greenhouse gas emissions (GHG). The targets covered emission of the six main GHGs, that is, Carbon dioxide (CO2), Methane (CH4), Nitrous oxide (N2O), Hydro fluorocarbons (HFCs), Perl fluorocarbons (PFCs) and Sulphur hexafluoride (SFs). In addition, the Kyoto Protocol also recognized the historical role that the industrialized countries have played in the emission of the greenhouse gas given their historical involvement in the industrial revolution and thus the moral responsibility that they carry in contributing to the global warming effects.

Ever since the entry into force of the Kyoto Agreement, the international climate change politics has been dealing with the question of who should pay the most and what responsibility should the traditional emitters and larger emerging developing countries bear in financing the effects of the climate change in countries and regions of the world such as Africa that are expected to be hit the earliest and hardest. As for Africa in particular, according to the IPCC, she is the region most vulnerable to the effects of climate variability and change because of her low adaptive capacity and because many Africans depend on rain-fed agriculture and natural resources for their existence^a^. The report went on to call upon the policy makers whether in Africa or elsewhere to draft policies and devise strategies aimed at tackling the effects of the global climate change in Africa and other vulnerable regions of the world. In regard to Africa, it highlighted the risks and dangers that tens of millions of Africans would face because of the increased water stress and spread of malaria if robust commitments and policies were not put in place to confront the effects of the climate change.

Moreover, through the UNFCCC, world community convened in Bali, Indonesia, in 2007 with the objective of seeking a comprehensive agreement on how to tackle the pressing issues of climate change. The result was that the participating governments to the conference adopted the so-called Bali Road Map (BRM) which included, among others, the Bali Action Plan (BAP). In short, the BRM was a set of decisions that participating governments considered to be the way forward to confronting the climate change threats. The BAP on the other hand, called on participating governments to adopt a `shared vision´ for a long-term cooperative action on climate change. Furthermore, two years after the Bali Conference, Copenhagen with the aim of agreeing to a global deal on the climate change. Unfortunately, the outcome of the conference was below everyone’s expectations. Furthermore, in the aftermath of the Copenhagen Summit, world community convened another conference in Cancun, Mexico, whereby the participating governments agreed on the establishment of a ‘Green Climate Fund’ and thus formalized the financial commitments made under the Copenhagen Accord. Finally, the recently held UN conference on the climate change in Durban, South Africa, was aimed at reaching agreements on the post 2012 Kyoto Protocol commitments. The outcome of the conference known as the *Durban Platform* agreed on a roadmap for a legally binding international agreement to combat the climate change by 2015 and be entered into effect by 2020. It also agreed on a series of commitments that are beyond the scope of this paper and therefore, not analyzed in depth.

Over the last two decades or so, there has been growing concern from the international community over possible security implications of the global climate change. The United Nations (UN), European Union (EU), United States (US), African Union (AU) and many other regions of the world have recently begun to ask how the security dimension of climate change will affect access to shared resources, national security, borders integrity, trade routes and international political alliances and economic relations. Against this background, the purpose of this paper is to examine the possible international security implications of climate change and discuss what the EU is doing in addressing possible threats of climate change on Europe itself. This will be done through the assessment of the initiatives that the European Union and Africa, South of the Sahara, have attempted to take in order to assist Africa to confront its adaptation and mitigation challenges of the global climate change while maintaining its primary goals of reducing poverty and implementing its sustainable development strategies.

The paper will discuss the Africa-EU Partnership on Climate Change and show what has been achieved to date. It will introduce the EU flagship instrument on climate change with Africa, namely, the Global Climate Change Alliance (GCCA), and present its innovative approaches. Finally, the paper will summarize the main points discussed and present challenges for the Africa-EU Partnership on Climate Change and offer recommendations to African and EU policymakers.

## Climate change and international security

Climate change has become one of the main concerns of the European and International politics. It poses serious risks and threats to the international and human security in many parts of the world, as well shown in the fourth assessment report released by the IPCC in 2007^b^. Before examining the security dimension of the climate change, a brief introduction is needed on what constitutes international security threat and how the concepts of human security and communities are defined. According to the report of the High Level Panel on Threats, Challenges and Change, international peace and security was defined as “any event or process that leads to large-scale death or lessening of life chances and undermines States as the basic unit of the international system^c^”. Based on this given definition, the panel identified six groups that constitute threats: interstate conflict; internal conflict; terrorism; nuclear, chemical and biological weapons; transnational organized crime; and economic and social threats including disease, extreme poverty, and environmental degradation. On the other hand, the concepts of the security of individuals and communities and national security are defined in the *Human Development Report* 1994 as “security symbolized protection from the threat of disease, hunger, unemployment, crime, social conflict, political repression and environmental hazards^d^”.

Based on the above- mentioned assessments of what constitutes threats as identified by the IPCC and the risks that they can pose to the international security, the UN, US, EU, AU and many major emerging powers such as China, India, South Africa, Brazil, and Russia started to look into the potential threats of the climate change on their national security, and to a larger extent, on international security very seriously. Thus, at the international level, since 2007, the UN has begun to mobilize international response and raise awareness on the implications of the climate change on international security. This began as a response to the acceptance of a concept paper circulated by the delegation of the United Kingdom of Great Britain and Northern Ireland to the Security Council members on April 3, 2007^e^.

The concept paper (S/2007/186) was aimed at asking the Security Council members to hold an open debate on the relationship between energy, security and climate. It was conceived by the UK delegation as part of the Security Council´s ongoing efforts to play a leading and constructive role in conflict prevention as stated in resolution 1625, adopted at a Council Heads of State-level meeting during the 2005 World Summit. The content of the concept paper goes as follows:

The UK concept paper places discussion of climate change within the resolution´s reaffirmation of promoting sustainable development as one of the broader strategies required to prevent conflict. The concept paper argues that increasing dependency on fossil fuels could result in climate change being accelerated, which would result in several types of impacts which combined might in turn increase the risk of conflict and insecurity. It identified the following wider implications of climate change and discussed their potential impact on issues closely associated with threats to international peace and security:

- Border disputes;
- Migration;
- Energy supplies;
- Other resource shortages, such as water, cultivable land and fish stocks;
- Societal stress; and
- Humanitarian crises.

In addition, this concept paper recognized the importance that the effects of the climate change could have on the environment; however, it directed the Council members to aim the debate on the security dimension of the impact of climate change. In so doing, it suggested that the Security Council members should debate and address among others, the following points:

- Prioritization of the risks climate change poses to international peace and security and the identification of new risks;
- The role the Security Council can play in an integrated UN approach to conflict prevention, including a greater emphasis on climate-related factors;
- The role of the Secretariat in better informing the Council and the wider UN membership of the security risks posed by climate change and the promotion of a more coherent response throughout the UN system.

Nevertheless, despite the positive acceptance of the UK paper by fellow Security Council members, many countries such as China, India and the Group of 77 (G77) rejected the idea of having to debate the issues of the climate change in the context of international security. They argued that the issues of the climate change should be dealt with by the General Assembly and the Economic and Social Council. From regional and national level perspectives, some of the widely accepted potential risks that climate change can create for the international security and some regions of the world are mass internal and cross-border migrations. As a response to this widely-held knowledge, many technical and policy studies have been commissioned to look into the situations in a comprehensive way. For example, according to the report by the Center for American Progress on Global Warning the Security Challenge of Climate Change, the “three regions in which climate-induced migration will present the greatest geopolitical challenges are: South Asia, Africa and Europe”.

Another report, among others, that looked into the threat of the climate change on national security is the panel of retired army generals assembled by the United States Center for Naval Analysis (CAN). The panel was asked to study the issue of climate change with regard to the national security of the United States itself. The report of the panel was released in 2007, and in it, climate change was defined as having the capacity to act as a `threat multiplier´^f^. That is, according to the report, climate change risks can exacerbate ongoing challenges and make untenable situations worse. Political stability could easily unravel if climate change risks are not addressed in timely manner. The said findings of the Panel received wider acceptance within the US policy making circles and in the international community as well. For instance, the current Secretary General of the UN Ban Ki-Moon agreed with the assessment of the Panel of the retired Generals while addressing the Security Council in 2007. In his speech he said that the concept of threat multiplier was “especially true in vulnerable regions that face multiple stresses at the same time- pre-existing conflict, poverty and unequal access to resources, weak institutions, food insecurity, and the incidence of diseases such as HIV/AIDS^g^”.

As regards to China, the Chinese position on the implications of climate change on national security can be categorized as official and non-official. That is to say, on the one hand, leading Chinese scholars and international relations experts such as Zhang Haibin of Beijing University have all recognized that climate change could pose a serious threat to the Chinese national security. Professor Zhang for instance, has made it clear that Chinese political leadership should consider the implications of climate change as a natural security matter beyond the traditional concept of security that they dearly hold. His views were further developed and argued in his 2010 book titled *Climate Change and China’s National Security*. Though the views of many of those experts and advocates are clearly influenced by the ongoing international debates on the climate security and the stance taken by the US, UN, EU and other developed countries on that subject, the Chinese government has not publicly accepted that position. Though it accepts that climate change is a serious issue that it needs to tackle with utmost urgency, the Chinese government nonetheless does not recognize it as a national security matter as do major developed countries. This is because for China or better said for official China, climate change is an economic issue that should be treated as a development challenge rather than strictly viewed as a security matter. This Chinese policy position was firstly expressed when the United Kingdom initiated a debate on climate change and national security in the UNSC in 2007.

Unlike the US and many other developed countries, China and several developing countries opposed the debate and the politicization of the climate change security at the UNSC level. The main point of their opposition was that the UNSC was not mandated to take upon itself the issues of global warming and that doing so would simply undermine other UN organizations such as the UNGA that are appropriate for it. Chinese Ambassador before the UN, Liu Zhimin, for instance, stated that “the developing countries believe that the Security Council neither has the expertise in handling climate change, nor is the right decision-making place for extensive participation”. At the same time, during the debate, Ambassador Liu, also pronounced what is the official Chinese government position as far as the climate change security debate was concerned. That is, he noted that “Climate change may have certain security implications, but generally speaking, it is in essence an issue of sustainable development.”

Therefore, the position of the Chinese leadership and government in Beijing has been all along to accept the urgency of the climate change as a non-traditional security matter but not strictly a national security issue as espoused by many Western and developed countries. This position is in fact shared by the highest Chinese leadership and expressed in international forums whenever possible. For example, during President Hu Jintao´s visit to the meeting on energy security and climate change in Japan in July 2008, Mr. Hu Jintao said the following:

“The climate change problem fundamentally speaking is a development problem, and must be comprehensively solved in the framework of sustainable development. International cooperation on climate change must start from correctly handling the triple relationship between economic growth, social development and protection of the environment, and must have guaranteeing economic development as its core, and strengthening sustainable development as its goal. It must have saving energy, improving the energy structure and strengthening ecological protection as its focus, and have scientific progress as a support, so as to continuously raise the capacity of international society to mitigate and adapt to climate change.”

Though the official Chinese leadership position on climate change and national security differs from the views advocated by some of its scholars and international relations analysts, it is worth recalling that China has significantly contributed to the debates on climate change internationally. One may even say, without any exaggeration that China and the EU have been at the forefront of the international leadership debate on the climate change. Hence, unlike the US, UK, EU and other developed countries, China’s international cooperation on the climate change security simply differs on what the Chinese leadership views as an urgent economic problem that needs urgent global solutions rather than a strictly national security as advocated by Western countries in general. It just refuses for its domestic and ideological reasons to see the direct link between the climate change implications and its national security imperatives.

Ideology and politics aside, it is worth noting that over the last years, China´s leadership has engaged the International Community with its food system. China has established a partnership on climate change with Africa, and has also considerably contributed to Africa´s capacity to adapting to and mitigating against the impacts of the climate change. As regards to the Climate Change and Environmental Protection challenges that Africa faces, Chinese government is committed to helping African countries enhance their capacity building in meteorological infrastructures and forest protection and management, and assisting them in disaster prevention, treatment of desertification, ecological protection and environmental management. China has also set up a dialogue and cooperation mechanisms on climate change with South Africa and several other African countries. For instance, the Hu Jintao´s Presidency even made it clear that it would actively promote China-Africa cooperation in climate change and assist Africa to address all the impacts of the climate change. As an example, it sponsored two study courses on the Clean Development Mechanism (CDM) aimed at improving the abilities of African and Asian developing countries to carry out CDM projects.

This policy approach is unlikely to change under President Xi Jinping given the importance that his Presidency has accorded Africa in his first overseas forays and policy declarations. On his first visit to Tanzania, the Republic of Congo, and on his way to the fifth annual BRICS Summit held in Durban, South Africa, in March 2013, President Xi reiterated China´s commitment to make Africa central in its foreign policy and provide it with assistance for its developmental challenges; and thus strengthen Africa-China strategic partnerships by providing aid, scholarship, and facilitating technology transfer towards the continent. Therefore, this internationalism with a special reference to Africa is a clear indication that China intends to deepen its cooperation and strengthen all aspects of its partnerships with Africa, and thus, compete with Europe as well as the rest of the world in its engagement with the continent.

At the European level, the EU has been at the forefront of the policy initiatives on climate change and its potential risks for many years. Over the course of the last decades, it has participated in various multilateral climate change negotiations and actively contributed in many of the international climate change policies. Hence, since the advent of the new millennium, the potential risks and threats of the climate change on the European security have taken prominent places in many of the major policy papers of the European Union. For instance, in the updated 2008 version of the European Security Strategy and the paper prepared by the EU´s High Representative for Common foreign and Security Policy (CFSP)^h^ and the Report on the Implementation of the European Security Strategy: Providing Security in a Changing World,^i^ climate change-related security threats on the European continent have become one of the key global issues on the EU international policy agenda. In effect, the EU views climate change as serious threats to its interests, and as a result of that, it has received wide support from the EU citizens in pursuing its overall climate change policies^j^.

Lastly, from the African perspective, the issue of climate change and its security implications are widely discussed by the government officials of respective African countries; and as a result, there is a growing awareness among the leaders of the continent on the subject. For example, the President of Uganda, Yoweni Musevini is famously quoted as saying that climate change is an aggressive act of the developed world against the developing world (Masood 2007). However, setting aside the recognition that African leaders grant to the climate change and its accompanying environmental degradations, they nevertheless perceive the security risk aspects of the climate change as another layer of mounting issues on top of the many existing challenges that they already face. That is, namely, the development problems, social unrest, conflicts over natural resources and civil wars, a burgeoning population, staggering youth unemployment, AIDS induced social crises and the capacity building challenges related to the attainment of the UN Millennium Development Goals (MDGs).

Henceforth, with all these challenges already present, the African leaders know that the security dimension of the climate change is something that is looming over the continent; nevertheless, they only seem to focus their time and energy on solving the pressing issues at hand. In fact, for many African leaders, they view the security risks of the climate change as additional global problems created by the developed world that simply need a concerted response from the international community see Jo-Ansie Van (2010)^k^. Yet, it is worth noting that for many of the African leaders, their pressing concerns about the security aspects of the climate change lie in the human security in Africa. That is so because of the direct consequences that that create in the areas of internal migration over natural resources and border conflicts and external migration that push the young and skilled Africans towards other African countries, Europe and other foreign destinations. Notwithstanding this, the African Union (AU) for instance, has debated the impacts of the climate change during the 8th Summit of the AU in 2007 and has as well taken the view that it regards climate change as a threat to the continent´s future well-being. However, what it has not done though is to institutionalize the security concerns of the climate change as a security concern as seen and perceived in the Western capitals.

The prevailing discourse of the climate change impacts on the international security is based on the traditional security rationale. That is, the notion of threats to the national security as understood by the State and the military establishments is well understood and widely discussed in the security literature. However, given that the potential impacts of the climate change are defined as non-traditional security threats, in order to truly understand the challenges of the climate change on the international security such as climate-induced migration, non-traditional security approaches are warranted. That is to say, the international community and the international organizations ought to shift their discourse of the climate-induced migration to the underlying socio-economic reasons that impel millions of vulnerable peoples to leave their homes and migrate. By doing so, one may understand the impacts that conflicts, war, poverty, and economic deprivation have on marginalized households, communities, and the rural poor. These above-cited factors can then be as compelling of a motive as the potential impacts of climate change on migration within a region of a given country or outside of it all together. This approach regards the socio-economic problems as the sources of many migrations even though the traditional security approach views the impacts of the climate change as the drivers for the mass-migration of millions of people originating in the areas affected by the climate change.

Therefore, the potential impacts of the climate change on the international security ought to be addressed by non-traditional security measures if peoples were made as the referent object of the discourse and analysis of the climate change. This view however, cannot be applied if the concept of the security is not broadened. That is, what is to be secured when addressing the challenges and threats of the climate change on the international security? Generally, as Lorraine Elliott pointed out, “the dangers and threats associated with climate change-induced migration are often articulated in terms of the possible detrimental impacts on the security interests of the United States, Europe and others”^l^.

With that said, the international community should link non-traditional security challenges such as climate change with other socio-economic problems as discussed-above in order to address the underlying sources of the human insecurity. That is to say, instead of addressing the challenges and potential threats of the climate change from the traditional security measures, i.e., military and defense responses, strategies that focus on sustainable economic development, social resilience mechanisms, land distribution and reforms, transparent system of property rights, gender equity policies, disaster risk management and localized food system should be promoted and integrated into national development policy plans.

### Climate Change and International Cooperation

Undoubtedly, climate change issues are global concerns that clearly warrant a concerted international response and cooperation. Therefore, the security implications of the climate change can only be effectively addressed if the international community tackles the said threats in a non-political and comprehensive way. That is to say, if well addressed by the international community, climate change can be a catalyst for better international cooperation. Developed countries and developing countries alike can map out multi-level strategies to successfully seek solutions for the various risk aspects of the climate change. For example, climate-induced migration, shared-resource conflicts, potential territory losses due to the rising sea-level will not be solved by any one country. Hence, they will all require international cooperation in order to effectively be managed. Doing so will without a doubt help the international community handle the security challenges of climate change at a multilateral level rather than leaving them to the resource-challenged and fragile states. In that respect, the EU has been advocating a multilateral response to the implications of the climate change on the international and regional security given that it considers it as a global problem. By making the climate change be recognized as a global problem, the EU has then intended to push for greater international support for climate change mitigation and adaptation, good governance and natural resource management, technology transfer and capacity building for crisis management of many developing countries. It also has equipped itself with an array of tools that can be used in its regional strategies such as the EU Neighborhood Policy, EU-Africa Strategy, and EU-Central Asia Strategy and EU-Middle East action plan in order to help assist countries deal with the impacts of climate change very effectively.

In the case of Africa for instance, the EU has undeniably various tools, *inter alia*, development policy instruments that it can use to assist Africa manage the impacts of the climate change on its security. It can do so within the context of the EU-Africa Partnership on climate change. As such, the EU has recognized that not doing so will inevitably have consequences on its international cooperation and of many African countries as well. Therefore, if the international community fails to grasp the importance of cooperation on the threats of the climate change, the international relations among many countries can consequently, be affected through forced climate migration, trans-boundary conflicts over shared resources and territory losses due to the rising sea. As a case in point, open border policy within the EU can be called into question and security concerns between several EU member countries could become an important policy problem for the Union and its 28 member states as well.

Climate change is considered by all countries today to be one of the pressing multinational issues or better said a serious “non-traditional security challenge” that requires global responses. Vulnerable developing countries and least-developed ones are expected to be hit the earliest and hardest. However, Africa, among all continents is said to bear the brunt of the climate change effects in terms of food security, sustainable water supply and extreme weather phenomena such as floods, drought and threats of desertification, according to the IPCC^m^. As an example, the economies of many communities, countries, and sub-regions of Africa, are already being hit by the devastating effects of the desert encroachment partly because of the climate change and also because of the land degradation caused by local practices. Consequently, developing countries, least-developed countries, as well as rich industrialized ones are all engaged in new partnerships and alliances with the intent to address the global challenges of climate change.

Hence, addressing those climate change problems that besiege many countries requires mutual trust and shared common visions among all countries of the world, i.e., EU engagement with Africa and Small Islands Developing States on climate change. In this respect, the EU and Africa, following the 11th Ministerial Meeting of the African and EU Troika^n^ that took place in Addis Ababa, Ethiopia, on 20 and 21 November 2008, jointly identified priority areas for action on the climate change that need to be addressed in order for Africa to address the adaptation and mitigation concerns of the climate change challenges. In addition to the identified priority areas for action as mentioned-above, the EU also launched the GCCA program whose main objective is to assist the LDCs and Small Islands Developed States (SDIS) adapt and mitigate the effects of the climate change challenges given their resources and technology constraints. In short, the following are the summary of the said priority areas for action in Africa-EU cooperation on climate change (see Ministerial Meeting of the African and EU Troika (2008) Africa-EU Declaration on Climate Change and Table 1): 1) Investment and financing possibilities in support of adaptation and mitigation initiatives in Africa; 2) Strengthening African capacities to better exploit opportunities under the carbon markets; 3) Water resources management and adaptation of agriculture; 4) Desertification, land degradation and scarcity of water; 5) Urban development; 6) Reduction of deforestation and degradation of the forests; 7) Sustainable management of firewood supply; 8) Access to energy and energy efficiency; 9) Sea level rise, small islands and deltas adaptation; 10) Development of renewable energy, notably solar in Sahara; 11) Support and cooperation on pollution inventories including GHG; and 12) Disaster risk reduction.

**Table 1:** **GCCA support to African national programs**

Country	Partners	GCCA Priority Areas	Sectors	Budget	Duration
**Benin**	UNDP, Ministry of Interior and Public Security, Ministry of Environment, Habitat and Urbanism, National Geographical Institute, National Remote Detection Centre, National Centre for the Management of Fauna Reserves	REDD	Forestry	Total value: €8.3 million (GCCA:€8 million and UNDP €0.3 million)	2012 to 2016
**Democratic Republic of Congo**	Ministry of Environment, Nature Conservation and Tourism, Congolese Institute for Nature Conservation, Centre for International Forestry Research	REDD	Forestry	Total value: €14.0 million (GCCA)	2012 to 2017
**Ethiopia**	Ministry of Agriculture and Rural Development, Environmental Protection Authority, DeutcheGmbh (GIZ), AgenceFrançaise de Développement (AFD),	Mainstreaming; adaptation; CDM	Overall development and poverty reduction	Total value: €13.7 million (GCCA, including €8 million EC fast start funding)	2011 to 2015
**Mali**	Ministry of Environment and Sanitation, Ministry of Foreign Affairs and International Cooperation	REDD	Forestry	Total value: €6.215 million (GCCA: €5.65 million; and Government of Mali: €0.565 million)	2010 to 2016
**Mauritius**	Ministry of Environment and Sustainable Development, Maurice Île Durable Commission	Mainstreaming; CDM	Overall development and poverty reduction	Total value: €3 million (GCCA)	2010 to 2013
			Energy		
**Mozambique**	Ministry for the Coordination of the Environmental Action, Ministry of Foreign Affairs Denmark (DANIDA)	Mainstreaming; adaptation; DRR	Overall development and poverty reduction	Total value: €47 million (GCCA: €15.2 million, including €5 million fast start funding from Ireland DANIDA: €31.5 million; and Government of Mozambique: €0.3 million)	2011 to 2015
**Rwanda**	Rwanda Natural Resources Authority, Ministry of Natural Resources	Adaptation	Land Management	Total value: €4.55 million (GCCA)	2010 to 2012
**Senegal**	Directorate for Environment and Classified Establishments of the Ministry of Environment	Adaptation	Coastal zone management	Total value: €4 million (GCCA)	2010 to 2015
**Seychelles**	National Climate Change Committee, Ministry of Home Affairs, Environment, Transport and Energy, Seychelles Energy Commission	Mainstreaming; CDM	Overall development and poverty reduction	Total value: €2 million (GCCA)	2010 to 2012
			Energy		
**Sierra Leone**	Forestry Division of the Ministry of Agriculture, Forestry and Food Security	REDD	Forestry	Total value: €5 million (GCCA, fast start funding from Ireland)	2012 to 2016
**Tanzania**	Ministry of Finance, Vice-President´sOffice- Division of Environment, Community Forest Pemba, Institute of Rural Development Planning, Sokoine University of Agriculture	Adaptation; REDD	Overall development and poverty reduction	Total value: €2.2 million (GCCA)	2010 to 2013
			Agriculture; coastal zone management; land management; natural resources; and water and sanitation		
**The Gambia**	Ministry of Finance and Economic Affairs, National Environment Agency, Department of Water Resources, Ministry of Forestry and the Environment	Mainstreaming; adaptation	Overall development and poverty reduction	Total value: €3.86 million (GCCA)	2012 to 2015
			Coastal zone management		
**Uganda**	Ministry of Water and Environment, Ministry of Agriculture, Food and Agriculture Organization (FAO)	Mainstreaming and adaptation	Agriculture	Total value: €11 million (GCCA, fast start funding from Ireland)	2012 to 2016

Source: European Union Commission, 2011: Global Climate Change Alliance.

The EU member states and their African partner countries are looking to tackle the climate change problems that they are facing by engaging and entering into alliances with partners that are willing to share their experiences and best practices on how to deal with energy security, environmental concerns, climate change and sustainable development challenges. Although Africa is supposed to be the most affected continent as cited by many studies in that respect and alluded to previously^o^ addressing the issue nowadays has become a paramount national security for all nations of the globe. Hence, climate change is one of the main issues alongside global security that many countries use to seek alliances and partnerships in international fora that offer political or technical support and assistance. The rationale behind entering into those alliances by many governments and policymakers in that matter is to tap into other governments and institutions that may provide valuable knowledge sharing, expertise and best practices towards the looming global warming threats. The European Union and Africa have become very active on the international scene in seeking strategic partners that share their common objectives through bilateral or multilateral coalition buildings. For example, they do so by increasing their voices, visibility, strategic alliances and partnerships within multilateral institutions such as the UNFCCC, the Organization for Economic Co-operation and Development (OECD) and the World Trade Organization (WTO).

In addition to seeking partners multilaterally as mentioned-earlier, the EU and Africa have also entered into bilateral joint strategic partnerships in order to enhance their political dialogue and deal with what they call issues of mutual concerns such as the climate change challenges. A case in point is the EU engagement in Africa through the *Africa-EU Strategic Partnership on Climate Change and Environment* and the support that the EU explicitly extends to the Climate for development in Africa program referred to as *ClimDev Africa*. This is an African initiative that seeks to make required information on climate change available to all African decision makers. Although the climate change challenge is no longer disputed by any country in the world, though the way to go about adapting to it and mitigating its effects and fully accepting its historic responsibility differs, the reality is that countries enter negotiations on climate change based on their national economic and security interests. That is so because countries in general base their climate change negotiation strategies on domestic interests driven by powerful stakeholders and local realities. This in effect is the reason why the United States government was lukewarm about the Kyoto Protocol and the Congress refused to ratify it. And as a consequence of that, former President Bush decided to withdraw the US from the parties of the Kyoto Protocol. Viewing this situation and understanding the politics of the International Climate Change Regime negotiations, one may be inclined to think that the leading countries in the EU, the US, Canada, China, India and other emerging powers as well as the African group on climate change negotiations and the Caribbean and Pacific Islands States representations should maybe focus on less contentious issues, and expend more energy and efforts onto the areas where divergence of positions are less considerable; i.e., the so-called `low-hanging fruit.´

The United States, EU, China, India, South Africa and Brazil considered as the leading climate change negotiators in the multilateral institutions should forge common positions on a given number of issues whereby showing concrete results in their efforts towards a common goal will incite the laggard and reluctant countries to follow their leadership. Doing so will create a momentum that the international community can seize upon to devise comprehensive international strategies to manage the global challenges of the climate change while adhering to the principle of “common but differentiated responsibility”. That is to say, to adopt clear strategies for the climate change adaptation, mitigation and the mainstreaming of climate-induced development challenges for the developing and least-developed countries into their sustainable development policies. Adopting this approach will certainly bring so many countries closer in their attempts to tackle their local climate change challenges. For, countries will be inclined to sharing their resources and technologies and best practices in adapting and mitigating climate change challenges for the greater global common good. For instance, the Pacific Islands and Caribbean Developing States that are more concerned of the rising sea-level threats can enter into alliances with advanced industrialized countries and gain tremendous experiences and knowledge and tap into their expertise on how to deal with those issues. The same can be said about the African countries as well. For instance, it is common knowledge that many African countries use fossil fuels as their main sources of energy for their electricity supply. Therefore, entering into strategic alliances with the industrialized countries targeting the issues of the climate change adaptation and mitigation can without a doubt help them acquire technologies that they need in order to successfully make the transition from fossil fuel energy policies to low carbon clean energy development through efficient technologies.

Another area where a strategic partnership can be of a greater benefit to Africa is the area of the Food-Based Dietary Guidelines (FBDGs). In Africa, many households and communities suffer chronic hunger, and as a consequence, millions of families are subject to nutrient-related diseases. China which has developed an impressive and respectable food system in the last three decades can share its expertise in that field and assist Africa to alleviate its nutritional problems. The Chinese Nutrition Society has been a member of the International Union of Nutritional Sciences since the year 1985. This membership and its extensive experience in community-oriented research in nutrition science is an asset that Africa can draw upon and strengthen its own locally-customized Food-Based Dietary Guidelines (FBDGs). China´s success in nutrition science has been remarkable in its achievements over the last three decades simply because it is mainly based on households and communities. That is to say, families and community-oriented research institutions are at the center of its food-based strategy. As M. L. Wahlqvist pointed-out, “a fundamental feature of the ability of China to play an early and foundation role in the development of the FBDGs in 1995 was that its nutrition scientists maintained a thread of basic community-oriented research during the most difficult times and rapidly re-asserted themselves once the opportunity arose^p^.” In essence, this operationalization is where cooperation with China can help Africa devise a strong and robust food system strategy that can help it address the problems of chronic hunger and nutrient-deficient diseases that many African families suffer.

To do so however, both China and Africa will have to devise a food system strategy that is inclusive of rural and urban communities and households (Wahlqvist 2009). That is to say, respective governments of the African countries and its Chinese counterpart have to put families, women and children at the center of their national food systems and health policies. In doing so, their food traditions and historical peculiarities (rural and urban) will be retained and the food systems that they will have put in place will reflect the local needs of their households and their communities alike.

## The threats and challenges of climate change on European and African Securities

From the scientific viewpoint, there is a widespread agreement based on technical studies that the African continent will be the hardest hit as a consequence of the climate change impacts. The potential threats on the security of Europe, i.e., the European Union and Africa are the subject of this section. The risks of climate change that are generally attributed to potential security implications fall into four categories: 1) the issues related to water access and scarcity; 2) the issues related to the decrease of food security for the general population; 3) the issues related to the climate-induced migration; and 4) the issues related to the impact of the climate change on poverty reduction and sustainable development and state incapacity (fragile states) to cope with the said challenges. When analyzing the issues related to the water access and scarcity in the context of climate change, there are three elements that need to be taken into consideration. First, there is a danger that climate change challenges can exacerbate the already existing water stress in Africa whereby water interdependence among many countries can easily lead to conflicts. Second, increased variability of waterfall due to the precipitation patterns, heavy rainfall at times and a lack of adequate infrastructure to capture and storage the water in many poor African countries can be a source of many social and economic unrest. Third, there is an assumption that water stress can directly cause conflicts among countries. However, this viewpoint is not at all conclusive because some studies show that water stress is not usually a cause of conflicts among states and, quite the contrary, it actually engenders cooperation rather than conflicts (Wolf et al. 2005).

Nevertheless, there are other studies that point out that sharing river basin can be a source of conflicts among countries (Gleditsch et al. 2006), and that water could also be the source of conflicts at a community level (WBGU German Advisory Council on Global Change 2007). As for the issues related to the decrease of food security for the general population in Africa, what many studies (CAN 2007 and WBGU German Advisory Council on Global Change 2007) have pointed out is the concern of a growing population on the African continent and the lagging agricultural production to keep pace with it, and the consequences that result from this disequilibrium; i.e., food shortages, undernourishment of millions of Africans and food security-induced migration. In addition, given that Africa´s agriculture production in many instances is tied to rainfall, any climate change variability could exacerbate the food insecurity situation if more arable lands were to turn into extensive dry lands.

Added to these challenges is a flurry of land deals and leases by international investors and foreign countries in Africa in the last ten years. For instance, the United Arab Emirates (UAE) has acquired 74,000 acres of land in the Sudan and intends to grow wheat, corn, potatoes and beans for its domestic population. China has also acquired seven million acres in the Democratic Republic of Congo (DRC) to produce palm oil for its own domestic consumption. India, South Korea and Malaysia are also very involved in land deals across Africa and principally in the Sudan, Ethiopia, and Madagascar. According to Brown (2011), in 2009, Saudi Arabia received its first shipment of rice produced on land it had acquired in Ethiopia. As such, these acquisitions of African lands add another layer of challenge to the existing food insecurity of millions of African households. However, many voices argue that this type of investment and projects will bring opportunities to Africa in its fight against the impacts of the climate change on food security. The opportunities here are understood as the transfer of modern technology and techniques of food production, and the improved infrastructure that will ensue. In contrast, other voices refute this assertion and point out that there is a danger that this production will not benefit Africans, and in the long-run this will prove counterproductive (Cotula et al. 2009). This viewpoint is also shared and well-articulated by Wahlqvist, McKay, Chang and Chiu in “Rethinking the food security debate in Asia: some missing ecological and health dimensions and solutions” (2012). They argue that, “our own view is that the long-term risks with such projects for African and poorer Asian nations may outweigh these potential benefits, particularly given the political realities and power relations in these developing nations. Also, the investing nations may generate resentment and accusations of continued forms of `colonial´ exploitation, when Asian nations would probably be better off attempting, to enhance their own level of internal food security^q^.”

It is widely known that Africa´s food security situation is critical. Out of 36 countries worldwide currently facing food insecurity, 21 are African (United Nations 2009). According to the same source (United Nations 2009), more than 300 million Africans are chronically hungry. This represents slightly more than a third of the continent´s population. And out of this number, Food and Agriculture Organization (FAO) (2008) estimates that at least 235 million live in sub-Saharan African countries. This, proportionately, makes sub-Saharan Africa among the regions of the world with a high number of chronically hungry people. Against this backdrop, Africa´s poorest and landless families, women, children and marginalized communities face a serious challenge to secure their food safety and security considering that their incomes have been falling due to the volatility of the food prices since the 2007–2008 international food crises. In light of this situation, African policymakers, international community, and the international organizations ought to reassess the prevailing approaches to tackling the impacts of the climate change on food security in Africa. That is to say, adaptive strategies such as reliance on foreign aid, NGOs, development agencies and global food trade as supported and promoted by major international organizations and developed countries. Though the above-outlined organizations and agencies have been providing responses to Africa as a whole in its fight against the impacts of the climate change, it is clear to any astute observer that the lack of coordination and policy coherence among those organizations makes their policy responses short of everyone´s expectations.

In essence, African governments should elaborate a broader and integrative climate change policy across the continent whereby food safety and food security should be a priority in each country´s national development strategy. In doing so, they can present a coherent African response to the international community and press the international organizations of global governance such as the EU, UN, G8 and G20, and present them with a single voice on what approach they deem effective to tackling the effects of the climate change on their food security. This integrative and broader response from African governments should however be locally based. That is, the policy approach should be designed to first address the food safety and food security of the localities, regional, and national communities, in order to develop resilient food systems across the African countries. The benefits of doing so will have implications beyond the challenges of the food security and its implications on peace and stability in the continent. As an example, this scenario was well pointed out by Wahlqvist et al. (2012) when arguing for the rationale and benefits of designing a resilient and sustainable food system in Asia. Hence, they said as follows:

“…the design of resilient and sustainable food systems is not just a matter of welfare and equity but could have major implications for the stability and peace of this key region^r^”.

This approach however, does not exclude the assistance of the international community or cooperation with the development agencies and NGOs that are specialized in addressing this issue. Resorting to those agencies however, should only be made in the time of distress or unforeseen natural disasters. Hence, redefining the concept of food security and adopting this purely locally based-solution against the impacts of the climate change could contribute to the development efforts and peace and stability of the region. Not doing so however, could prove to be a bigger obstacle against the African development strategy and economic gains. Thus, as Mark L. Wahlqvist, John McKay, Ya-Chen Chang and Ya-Wen Chiu have asserted, “as populations increase and the full impacts of climate change are felt, competition for the scarce resources of food and water has the potential to destabilize the region and undermine the many development gains that have been achieved in recent years” (Chellaney 2011; McDonald 2010; McKay 2009)^s^.

**Figure 1:**
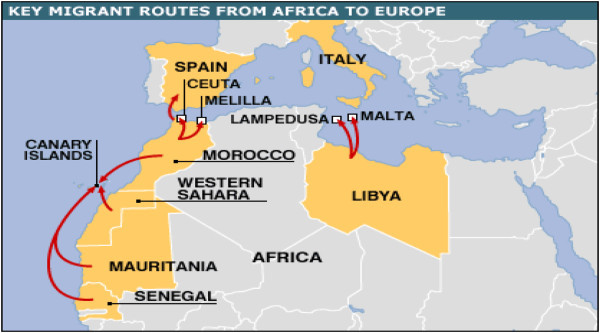
**Spain vows to curb migrant wave.** Source: http://news.bbc.co.uk/2/hi/europe/5313560.stm.

**Figure 2:**
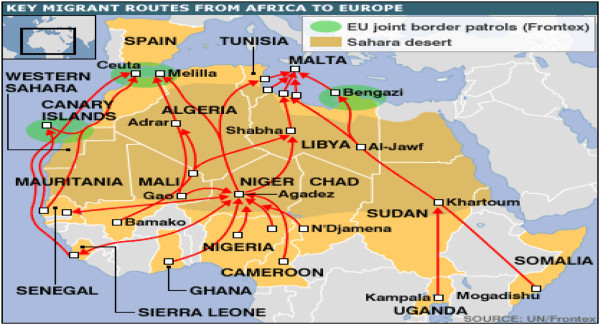
**Key facts: Africa to Europe migration.** Source: http://news.bbc.co.uk/2/hi/europe/6228236.stm.

In respect to the climate-induced migrations, there could either be an internal migration or external migration due to the risks and threats of climate change. First, affected populations by the variability of the climate change may just choose to move from their natural habitats to a different region within the same country. This unfortunately, could create tensions and conflicts over resources of the host region. When this phenomenon is extended to other countries in the region, it can then easily blow into a major regional crisis where displaced populations become refugee crises victims. Second, it is also argued that climate-induced migration could be the result of sea-level rise and flooding that would displace many people and incite them to look for new life opportunities in distant lands. And in the case of many Africans and South Asians, the destination is usually Europe while the northern part of the continent is used as a transit region before arriving at the target one (see Figures 1 and 2 key migrant routes from Africa to Europe). This international migration however, is seen and perceived as generating security concerns not only in the transit countries but also in the target areas. In this case, the security threats are to be in the European countries because they are the ones taking in thousands of immigrants and dealing with all the ramifications of what that entails.

The implications of the threats by climate change on the European security are manifold. However, in this paper, we focus our analysis on two of the main climate change risks on the European Security, i.e., the European Union. These two security risks are namely: climate induced migration to the European shores from Africa and Asia and potential risks on the Energy supply route to the European countries.

First, the risk that induced climate migrants from Africa and Asia can pose to the European security is exacerbating the already existing tension between the many immigrants in Europe and the native populations. For example, in many major European Union countries such as Germany, France, the Netherlands, Italy and Belgium, there is constant tension between Muslims immigrants and their not well integrated children and the non-counterpart native populations. Therefore, if the induced-climate migrants were to be Muslims or otherwise in a substantial number, for example, this could easily raise the level of existing tension and potentially fuel anti-immigrants, anti-Muslims feelings just like what we have witnessed over the recent years in France, Denmark and the Netherlands. Consequently, this can strengthen the nationalist anti-immigrant backlash that is growing in many European countries. Further, if this potential risk is coupled with the current economic situation in many EU countries such as Spain, Portugal, Greece, and Italy that have opened their doors to millions of immigrants in the last decade or so, the prospect of not managing well the issue of increased immigration could have direct consequences on the EU internal cohesion itself

Second, climate change potential security risks on Europe could also come from the climate impacted energy producing and exporting countries to the European market. That is, if countries such as Nigeria, Algeria, and Equatorial Guinea among others were to be severely affected by climate change, gas and oil supply and distribution into the European market could easily be disrupted. In this scenario, many European countries will face economic and security problems because oil prices will likely rise and that can have negative impacts on economic growth. In addition, this potential energy security risk will not just be confined to Europe; the risks will be shared worldwide. Therefore, we believe that the EU should do its utmost to integrate its diverse foreign policy commitments to Africa and make them more coherent in order for the Partnership on climate change to not encroach on other policy areas. Moreover, we also believe that the EU and its African partners should engage all development actors operating in Africa to incorporate and link climate change issues in their development projects. However, it is worth noting that in order for that strategy to be effective, it has got has to clearly be in tune with the poverty reduction policies in place aligned with the Millennium Development Goals (MDGs).

Third, we also believe that the African leaders themselves should address climate change as a development issue and institute policies that counter its major impacts on human development and sustainable economic growth. In sum, we consider that once those recommendations that we have proposed above are taken into account by the African and the European policymakers alike, the Africa-EU Partnership on Climate Change will be a robust policy that both sides can use to combat the adverse effects of the climate change in Africa and progressively help her transition to low carbon economy.

Furthermore, as regards to the security implications of the climate change on fragile states in Africa, the concern is that impacted fragile states might easily disintegrate and that could have untold security consequences across the continent and beyond. That is, given that many of the African states are already struggling to provide basic state functions to their citizenry, an additional security issue emanating from the climate change may increase instability and make matters worse for those failing states.

### EU-Africa´s strategic alliance and climate change

The leadership of the European Union in climate change diplomacy is widely recognized and accepted within the international community (Pinder and Usherwood 2007). The EU has been at the forefront of the negotiations to address and combat the climate change challenges at the international level. And at the same time it has been engaging other regions of the world such as Africa and the Caribbean Islands Developing States and their Pacific Islands counterparts in its quest to reach a binding and accepted multilateral agreement on the climate change threats and challenges. From the political viewpoint, on the one hand, it has been one of the main promoters if not the principal promoter of the global dialogue within the UNFCCC. On a strategic level, on the other hand, the EU has also played a major role in setting the agendas of the climate change negotiations at the international and global level. In the last quarter of a century, the European Union has been one of the leading voices in setting the agendas to combat the global climate change threats in the international forums; as noted above. This agenda setting in the international climate policy has been possible because of policies enacted domestically within the EU. That is to say, policies agreed on by the Commission and the member states (EU Climate Change Policy is a shared-competence policy (Art. 4 (2e) TFEU) devised to combat the climate change threats. For example, the six Environmental Action Programme, approved by the Council and the Parliament, contained a ten-year framework for promoting sustainable development in the fields of climate change, nature and diversity, environment and health, and natural resources and waste, The result of that has been the prioritization of the climate change as a policy area within the overall strategy of the EU sustainable development policy. As a case in point, with the approval of the sixth environmental action program, in that same year, the EU played a leading role in the World Summit on Sustainable Development in South Africa (Pinder and Usherwood 2007).

During the negotiations leading to the signing of the Kyoto Protocol, the European Union clearly played a leading role in shaping the positions of the group of the Sub-Saharan African Countries. Its proposal on a `differentiation mechanism´ based on the principle of *common but differentiated responsibilities* convinced many African countries and larger developing countries alike to follow its lead and agree with it during the KP negotiations. That was so because the rationale behind the `differentiation mechanism´ simply recognized the historical contribution of the industrialized countries in contributing disproportionately to the emission of the carbon dioxide (CO2) and other greenhouse gases. And on the contrary, the mechanism did not require that the developing countries and in particular African countries whose contributions to the global greenhouse emissions have been historically trivial cut their emissions, since doing so would adversely affect their economic growth and developments. At the same time, the European Union proposed that the Kyoto Protocol set the framework or guidelines through which developing countries could use alternative means of production that would be environmentally friendly, to say the least.

Moreover, as a sign of leadership in addressing the global climate change challenge, the EU unilaterally made a commitment under the Kyoto Protocol to achieve at least a 20% reduction of its greenhouse gas emissions (GGE) by 2020 compared to 1990 levels. In addition, the EU also endorsed an objective to reduce its GHG emission by 30% as its contribution to an overall global and comprehensive agreement for the post-2012 period. This endorsement is however, incumbent upon an equal commitment by other developed countries to reduce their GHG emissions and a commitment also by the economically more advanced developing countries to do likewise in accordance to their responsibilities and respective capabilities^t^. By doing that, it set the agenda for the international community at large and the African continent in particular to accept the search for a more effective and decisive action to confront the challenges of the global warming while recognizing and accepting the principle of *common and differentiated responsibility*. This principle however, did not impede countries from carrying-out their fair share of responsibility to limit the global temperature increase to no more than 2°C.

By recognizing the climate change challenges as a foreign policy concern, the EU came to the realization then that it would effectively advance its climate change policy agendas on a global level if it engaged Africa as a strategic partner via enhanced political dialogue. This is in contrast to the uncooperative stance that the United States government has taken against the ratification of the Kyoto Protocol. This realization resulted in an enhanced Africa-EU Partnership on climate change, drafted into the 2007 joint-Africa-EU Strategy (JAES) document. Thus, this would be the instrument that both continents would use in their cooperation to tackle their energy security challenges and environmental concerns, among which the climate change is of the highest priority. The Africa-EU Strategic Partnership and a Joint Africa-EU Strategy document came about as a reaction to what Europe and Africa perceived to be a solution for the new threats and issues that required joint responses and coordinated efforts based on common vision and shared interests.

The Partnership on climate change is designed to provide (a) a platform for dialogue, cooperation and exchange for tangible measures to respond to climate change between Africa and the European Union; (b) to have close links to the GCCA and the ClimDev Africa Programme (ClimDev); and (c) to represent an integrated framework for Africa-EU co-operation on climate change. The partnership has two priority actions and each priority has its own objectives. That is, the first priority action is defined as `common agenda on climate change policies and co-operation**´** and its objectives are as follows: 1) “enhanced dialogue, and common approaches, including at multilateral level, on climate change challenges in Africa, Europe and globally, in particular in view to the negotiations for a global and comprehensive post-2012 climate agreement”; 2) “strengthened capacities to adapt to climate change and to mitigate its negative effects”. Furthermore, according to the Partnership, the following were the expected outcomes of the joint policy:

1) a strengthened Africa-EU dialogue on the development, implementation and further improvement of climate change related initiatives and treaties, in particular in view of the negotiations of a global and comprehensive post-2012 climate agreement; 2) systematic integration of climate change into African national and regional development strategies as well as into Africa-EU development cooperation; 3) increased capacity in African countries to adapt to climate change and mitigate its negative effects, including through climate risk management and resilience to deal with climate-related disasters; 4) improved data, analytical methods and infrastructure for sectoral Climate Risk Management (CRM), monitoring climate variability and detecting climate change with strengthened observation networks and service in centres in Africa; 5) reduced rates of deforestation and better preservation of forest ecosystems, while improving the livelihood of forest dependent populations; 6) increased benefits for Africa from participation in the global carbon market and enhanced capacity of African negotiators in the international market; and 7) increased energy efficiency and resilience to climate change in the African economies.

**Figure 3:**
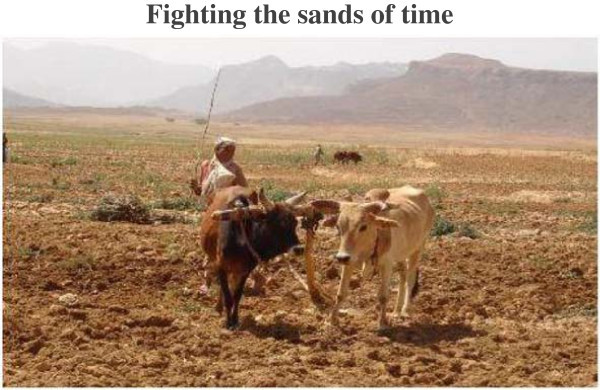
**Fighting the sands of time.** Source: http://www.africa-eu-partnership.org/successstories/fighting-sands-time.

The second priority action is defined as `cooperation on land degradation and increased aridity, including the Green Wall for the Sahara Initiative´ and its principal objective is as follows: to “combat desertification and improve the livelihoods of the inhabitants of the countries of the Sahara and Sahel zones of Africa (see Figure 3 as an illustration of land degradation in Africa). “The outcome that was expected from the second priority action in accordance with the Partnership is a described below:

1. Progress towards reversal of desert encroachment and soil degradation;
2. Improvement of micro-climatic conditions and reduction of land degradation.

Hence, the extracted statement below from the Africa-EU-partnership website and the picture shown in the following page further explain the aims of the GGWSSI projects in Africa.

*“A number of cross-sectoral actions are underway to address desertification/land degradation and other pressing environmental issues in the circum Sahara and Sahel zones, such as climate change adaptation, water shortages and biodiversity loss. This African regional framework programme, entitled the Great Green Wall of the Sahara and Sahel initiative (GGWSSI), aims to tackle both environmental and poverty-related challenges.”*

In addition to the objectives, outcomes and priority actions of the Africa-EU Partnership on Climate Change as described-above, the partnership also aimed at making the African populations its primary beneficiaries while reducing the impact of climate change on their environment. The partnership particularly seeks to enhance the sustainable land management in order to help increase growth and improve the livelihood of the African rural populations. In concrete, the African farmers and most vulnerable segments of the population, with limited access to water and victims of the food price volatility and insecurity are the primary targets of the Partnership. The Partnership involves various important stakeholders from the UN, Africa and Europe in order to effectively and positively contribute to its success. The main actors behind it are the African Union and the European Union Member States; the African Union Commission and its European Union counterpart; the African Development Bank (AfDB); the United Nations Economic Commission for Africa; the African Regional Economic Communities (RECs) responsible for environmental management issues and the Civil Society Organizations from the two continents. As of today, a considerable amount of progress has been achieved in certain areas where the partners have been working together even though the Partnership is only 3 years old. Examples of the achievements can be cited as follows:

“Enhanced political dialogue and cooperation predominantly under the EU Global Climate Change Alliance; reinforcement of African negotiator´s capacities in international climate negotiations; strengthening African possibilities to better exploit opportunities under the carbon market; water resources management and adaptation in the field of agriculture; and sustainable land management, fight against desertification and avoiding deforestation.”

In addition to the progress noted- above, the Partnership has made concrete inroads, in that we can state the following:

“the joint-EU-Africa declaration on climate change adopted on December 2008 in Addis Ababa has provided concrete opportunities for cooperation and dialogue in relation to the negotiations in the run-up to the Copenhagen Conference. This is continuing in the run-up to the Cancun Conference (November 2010) and beyond; the channeling of € 45 M of EU financial support in the period 2008–2010 under the Global Climate Change Alliance Initiative (GCCA). This financial support has been already provided to six African countries (Mali, Mauritius, Rwanda, Senegal, Seychelles and Tanzania). The programming of the GCCA 2010 interventions in Ethiopia and Mozambique is currently being finalized; GCCA interventions in Africa support the implementation of National Adaptation Programmes of Action (NAPA) Priorities fostering climate resilience in the water management, rural development and agriculture sectors and enhancing institutional capacity to mainstream climate change into policy, regulatory and strategic development planning; a contribution of € 8 M has been provided in 2009 to the ClimDev Africa flagship initiative and to the AU Commission climate change and desertification coordination efforts; and preparation of the Great Green Wall for the Sahara and Sahel Initiative implementation phase through cross-sectoral actions aimed at sustainable management of natural resources.”

As previously stated, the Africa-EU Partnership on Climate Change has two priority actions with each carrying its own objectives (see notes above). The two main flagship initiatives of the partnership are the GGWSSI, which was initiated and led by Africa and the GCCA, which was the European Commission initiative designed to assist mainly the LDCs and SIDS and aimed at the sustainable management of their natural resources.

Thus, from 2008–2010, the Commission and the Member States made available around € 100 million from the EU budget and bilateral contributions for vulnerable countries. In addition, the Commission made available another € 40 million as part of the 10th European Development Fund (EDF) for the promotion of regional approaches and assistance in the ACP grouping of countries. As such, it was expected that by 2011, the GCCA initiative would support activities in many of the vulnerable countries. The African countries that have been provided financial support through the initiative are Mali, Mauritius, Rwanda, Senegal, Seychelles and Tanzania. Ethiopia and Mozambique have been selected to benefit from the initiative as well; but as of time of writing, they are pending the finalization of the process. Each beneficiary country will receive a country-tailored support on climate change issues and an enhanced political dialogue with the EU as stipulated into the Africa-EU Joint Strategy. For further details on the GCCA, see Table 2 below.

**Table 2:** **The selected countries that benefited from the GCCA support in 2008, 2009 and 2010**

Beneficiary Country	Year of Commitment	Type of Contract	Project Duration	Contract Signature	Sector of Intervention
Tanzania	2008	Financing Agreement with the Government of Tanzania	2010-2013	16/06/2010	Eco-villages Agriculture and land use Natural Resource Management
Mali	2009	Financing Agreement with the Government of Mali	2011-2016	7/12/2010	Mainstreaming CC Forestry Gov. Capacity Building
Mauritius	2009	Financing Agreement with the Government of Mauritius	2010-2015	03/08/2010	Sustainable Development Mainstreaming CC
Rwanda	2009	Financing Agreement with the Government of Rwanda	2010-2013	12/05/2010	Land use
Senegal	2009	Financing Agreement with the Government of Senegal	2010-2013	19/10/2010	Coastal Zone Protection
Seychelles	2009	Financing Agreement with the Government of Seychelles	2010-2013	14/07/201	Sustainable Development Energy and CDM
Ethiopia	2010	N/A			Mainstreaming CC Gov. Capacity building Water and Agriculture Land Management
Mozambique	2010	N/A			Gov. Capacity Building Raising Public Awareness Agriculture and Land Use

Source: http://www.gcca.eu/cgi-bin/view.pl?&page=41&lg=2&url_content=GCCA-Beneficiaries.

The third Africa-EU Summit was held in Tripoli, Libya, in November 2010. During that Summit, the Heads of State and Government of Africa and the European Union reiterated their commitments to maintain the Joint Africa-EU Strategy as the framework for their future cooperation and adopted as a result, the second Action Plan 2011–2013 as their firm commitment to pursuing the realization of their Partnership. They recognized the pressing issues that the world was facing at the turn of the last decade and called for the reaffirmation of their partnership to address them. *Those issues were stated as tackling the effects of climate change, conflict prevention, good governance, achieving a sustainable energy market including investment particularly in renewable energy resources, providing food security, achieving the Millennium Development Goals, combatting HIV/AIDS and addressing the realities and challenges of migration and its links to development*.

The Summit concluded by calling on the Partnership to specifically focus on the five components of the 13 priority areas identified by the Joint Africa-EU Joint Group on Climate Change in November 2008. The deliverable initiatives from the five components identified as priority areas of the Partnership are “the Great Green Wall of the Sahara and the Sahel Initiative (GGWSSI); the CLIMDEV and AMESD programs; the activity of building and enhancing African negotiators´ capacity in negotiations under the UNFCCC; the GCCA and the fight against deforestation^u^.” It is worth noting that the Africa-EU Partnership on Climate Change second Action Plan addresses disparate issues from cross-cutting areas. One project that is worth mentioning as part of the capacity building, though initiated from the first Action Plan, emanating from the Africa-EU Partnership in favor of sustainable development is the Congo Basin Countries Capacity Building on GHG Inventories. This type of training initiative is a concrete example of how the EU assists African countries to build their capacity in taking stock of their emission and effectively participate in the international negotiations relating to the climate change governance. In particular, the Partnership for Forests of the Congo Basin provides support and capacity building to the member countries of the Commission of Forests of Central Africa in international negotiations over climate change. The partnership aims at assisting those countries in the calculation of GHG emissions and providing them with training on the so-called mechanism for Reduction Emissions from Deforestation and Degradation in developing countries (REDD). Hence, the Africa-EU Partnership is only three to four years old, but the intensity and scope of the Partnership has been present throughout the latest international climate change negotiations, i.e., the Copenhagen and Durban Conferences.

## Challenges, implications and policy recommendations

There are many challenges to the Africa-EU Partnership on Climate Change that need to be addressed by both partners if the partnership is to have a long-lasting success despite what it has achieved so far. It is true that the Partnership is in its early operational phase, but one can already see that the main actors that are driving the partnership are still the official institutions, be they African Union Commission and the respective African States or the European Union Commission and its Member States. The partnership needs to be more people centered rather than institutionally driven in order for it to have a real impact on the people’s lives. Besides, we believe that the partnership will have wide range positive effects if the private sector and academia are called upon to fully participate in addition to fostering the full involvement of the civil society organizations. Also, the European Union needs to sort out the issues of financing and earmark funds that can specifically be used for the purpose of combatting climate change. In doing so, it will avoid using finances from the EDF that are earmarked for development projects in programs and areas that address climate change issues. That is so because for Africa, development concerns are still a priority even though it is clear that climate change adaptation and mitigation are to be part of its overall sustainable strategies if it is to transit to the low carbon green economy.

Moreover, other impediments of great importance that in our view need to be addressed by both parties are the apparent EU policy incoherence in the fields of energy, trade and agriculture and the lack of resources by the African side when it comes to disbursing local funds to tackle climate change challenges. On the EU side, it is worth remembering that the Environmental Policy of the Community is a shared policy, that is, the Commission and the EU Member States share powers in decision-making related to the climate change towards third countries. Thus, this is a clear challenge to the Africa-EU Partnership on Climate Change. However, it is also important to recognize that the EU has made important progress in streamlining its incoherent policies by adhering to the Paris Declaration on Aid Effectiveness and attempting to mainstream the climate change issues by linking them into its development cooperation policy strategies towards Africa. On the Africa side, there is a myriad of challenges that need to be addressed if adapting and mitigating the effects of the climate change were to be effective. Chief among them, in our view, is providing capacity development to all African stakeholders when dealing with everything climate change, whether at governmental or non-governmental level. This is in addition to the financial challenges that Africa clearly faces when addressing climate change problems considering that development challenges are the primary concerns of the African governments and their leaders.

Furthermore, another significant challenge that the Africa-EU Partnership on climate change faces in our view is how to circumvent the growing influence of emerging powers such as China, India and Brazil in the international climate change politics. That is to say, despite the strategic nature of the partnership, Africa is of the view that the parties that have historically contributed to the global warming effects pay the most. This assertion is clearly the position of many developing countries and especially the larger ones such as China, India, Brazil and South Africa. Consequently, this can limit the scope of the Africa-EU Partnership on climate change and make the EU less influential on its African partners despite the solemn declaration of the partnership. As a case in point, during the Copenhagen Conference on Climate Change the EU clearly struggled to have full African support behind it because Africans clearly sided in their policy approach with the above-mentioned emerging countries.

## Conclusions

The likelihood that climate change risks can affect the international security is hotly debated in recent years by the international community. The United States, EU and many other countries have commissioned studies to look into the potential impacts of the climate change on their national security. The European Union, over many years, has taken a leadership role at fostering international cooperation in seeking to tackle the climate change threats through its diverse policy tools and initiatives with disparate regions of the world. The EU is particularly weary of spill-over effects of climate change threats whereby induced-climate migrations from third nations can have incalculable consequences on its territory. In that regard, it has developed specific policies to address the impacts of climate change in many developing countries and foster greater international cooperation in that respect. By recognizing that the threats of the climate change are multidimensional and no one country alone can address them effectively, the EU and the African Union have adopted joint-policy initiatives to tackle the issues of climate change.

This reality and many other issues of global concerns brought the European Union and Africa to enter into strategic partnerships and adopted the Joint Africa-EU Strategy as a guiding document to tackle many issues of common concern, *inter alia*, the Africa-EU Partnership on Climate Change. Given what has been accomplished so far in terms of concrete policies and deliverables based on the Action Plan 1 & II of the Joint Africa-EU Strategy, the Partnership on climate change between the two continents can be considered a success considering that it has only been in operation for five years. However, this does not mean that there are no issues of concern and disagreements between the two parties that are not worth addressing in order to make the Partnership more effective and inclusive. For example, issues such as the incoherence of the EU policies in various fields towards Africa have been of great concern for both parties in their view of the Africa-EU Partnership on climate change.

In addition, on the African side in particular, challenges regarding the capacity building on climate change adaptation and mitigation have also been a great concern in its partnership on climate change with the EU. Last but not least, both sides have come to recognize that concerns on the climate change should not supersede the development and poverty reduction goals of the African continent. However, the challenge is how to link climate change issues and development issues in a balanced and coherent manner that is beneficial to Africa and responsive to its international climate change obligations.

## Endnotes

^a^See The Intergovernmental Panel on Climate Change. Working Group II contribution to the Fourth Assessment Report: Impacts, adaptation and vulnerability, http://www.ipcc.ch/working_groups/working_groups.shtml.

^b^See Intergovernmental Panel on Climate Change (IPCC), Fourth Assessment Report.

^c^See United Nations 2004 High Level Panel on Threats, Challenges and Change, A More Secure World: Our Shared Responsibility.

^d^See UNDP, Human Development Report 1994 (Oxford University Press, 1994).

^e^See UN Security Council Letter dated 5 April 2007 from the Permanent Representative of the United Kingdom of Great Britain and Northern Ireland to the United Nations addressed to the President of the Security Council.

^f^CAN 2007, National Security and the Threat of Climate Change, Military Advisory Board.

^g^See Security Council Debate on Climate Change as a threat to International Peace and Security, 17 April 2007

^h^See European Council (2008a) `Climate Change and International Security: Paper from the High Representative and the European Commission to the European Council´. S113/08, 14 March.

^i^See European Council (2008b) `Report on the Implementation of the European Security Strategy: Providing Security in a Changing World´. S407/08, 11 December.

^j^See Special Euro Barometer 300, Europeans´ attitudes towards climate change, September 2008.

^k^See The African Union's response to climate change and climate security, Jo-Ansie Van Wyk, Institute for security studies, Pretoria.

^l^See Elliott et al. 2012. (Eds.), Climate Change, Migration and Human Security in Southeast Asia (Singapore: S. Rajaratnam School of International Studies (RSIS), p*.*5.

^m^The Intergovernmental Panel on Climate Change. Working Group II contribution to the Fourth Assessment Report: Impacts, adaptation and vulnerability, Cambridge University Press. 2007.

^n^The EU Troika here refers to the EU foreign ministries holding the EU Presidency, and the country that will hold the Presidency next, the European Commission and the EU Council´s general secretariat.

^o^;See The Mutual Review of Development Effectiveness in Africa: Promise & Performance. A Joint report by: the Economic Commission for Africa and the Organization for Economic Co-operation and Development 2011.

^p^See Wahlqvist (2009) in Connected Community and Household Food-Based Strategy (CCH-FBS): Its Importance for Health, Food Safety, Sustainability and Security in Diverse Localities.

^q^See Wahlqvist et al. (2012) in Rethinking the food security debate in Asia: some missing ecological and health dimensions and solution.

^r^Ibid. p. 667.

^s^Ibid. p. 667.

^t^See Africa-EU Declaration on Climate Change during the 11th Ministerial Meeting of the African and EU Troikas. Addis Ababa, 20–21 November 2008.

^u^See JAES Action Plan 2011–2013 Partnership on Climate Change and Environment.
